# A new tool for mini-open carpal tunnel release – the PSU retractor

**DOI:** 10.1186/1471-2474-9-126

**Published:** 2008-09-22

**Authors:** Sunton Wongsiri, Porames Suwanno, Boonsin Tangtrakulwanich, Varah Yuenyongviwat, Ekkarin Wongsiri

**Affiliations:** 1 Department of Orthopaedic Surgery and Physical Medicine, Faculty of Medicine, Prince of Songkla University, Hat Yai, Songkhla 90110, Thailand; 2 Department of Electrical Engineering, Chulalongkorn University, Bangkok, Thailand

## Abstract

**Background:**

Mini-open carpal tunnel release has become increasingly popular for the treatment of carpal tunnel surgery. The main advantages are shortening recovery time and return-to-work time. However, the risk of neurovascular injury still remains worrisome.

**Methods:**

In this study, we developed a new retractor (herein called the PSU retractor) modified from the widely used Senn retractor, with the aim of decreasing the risk of neurovascular problems from normal procedure. 3-Dimensional computer design software (SolidWorks^® ^Office Premium 2007 SP3.1) was used to construct a 3-D PSU retractor prototype. An amputated arm from a 30-year-old woman diagnosed as synovial sarcoma at the shoulder was used to test the maximal visual length. A mini-surgical incision was performed at 3 cm distal to the transverse wrist crease and a tiny flexible ruler was inserted through the tunnel beneath the skin to measure the maximal visual length.

**Results:**

Our new retractor showed significantly better maximal visual length compared to the Senn retractor (47.7(8.1) mm vs. 39.2(6.5) mm). In addition, most assessors expressed a higher satisfaction rate with the PSU retractor than with the Senn retractor (7.3 (1.9) vs. 6.3 (1.1)).

**Conclusion:**

In conclusion, we have developed a promising new retractor using a computer design program, which appears to be an improvement on the currently available equipment used for mini-open carpal tunnel surgery. However, further clinical studies are needed to confirm our initial findings.

## Background

Carpal tunnel syndrome (CTS) is the most common nerve entrapment problem faced by orthopaedists. Surgical treatment is generally recommended in cases which fail to respond to conservative measures. Surgical treatment [1] normally involves cutting the transverse carpal ligament to reduce the carpal tunnel pressure. Open carpal tunnel release (OCTR) remains the accepted standard operation [2], but newer procedures include endoscopic or semi-blind mini-incision techniques are being increasingly performed. Postoperative pain and late recovery are the main drawbacks to OCTR [3-6]. Mini-incision carpal tunnel release using various types of tool such as the Indiana Tome (Biomet, Warsaw, USA), the KnifeLight (Stryker Instruments, Kalamazoo, Michigan, USA) and the "Safeguard" system (KMI, Inc., San Diego, USA) have recently been gaining popularity and are aimed to reduce the problems that result from this open surgery. However, these newer techniques cannot allow the surgeon to fully visualize all important structures and thus there is a somewhat increased risk of neurovascular injury to the median nerve, the recurrence branch of the median nerve or the superficial palmar arch artery [7,8]. In order to avoid such potentially preventable complications, an instrument to assist in mini-incision techniques would be of benefit. Recent advances in computer-assisted design programs, such as SolidWorks, are very useful in designing and developing new instruments, including medical instruments, and are commonly used for 3-dimensional tool design by engineers. These programs provide animation together with mechanical testing within the program. In this study, we demonstrate the method we used to develop a new instrument for mini-open carpal tunnel release and also show some preliminary results of using this new retractor, we call the PSU retractor.

## Methods

### Design and development of PSU retractor

**Figure 1:**
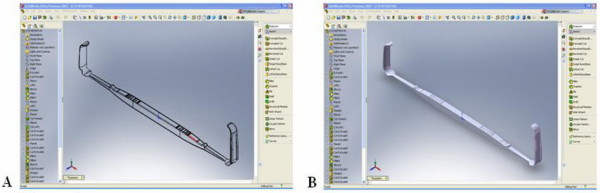
**3D model of PSU retractor**. 3D model of PSU retractor has created by SolidWorks program (A), (B).

**Figure 2:**
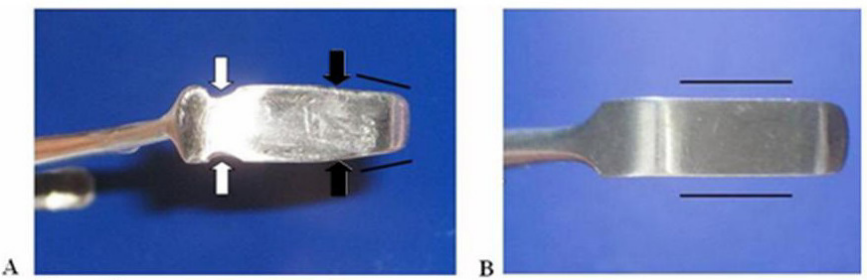
**Bilateral concave and taper blade of PSU retractor**. PSU retractor (A) has bilateral concave (white arrow) and taper blade (black arrow) for aid insertion compared to Senn retractor (B).

**Figure 3:**
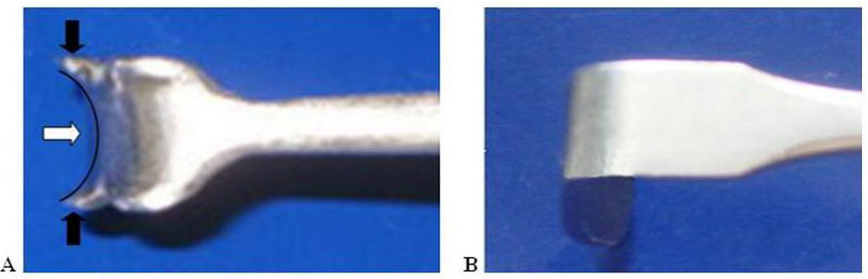
**Concave curve with bilateral wing of PSU retractor**. Demonstrated concave curve (white arrow) at distal end with bilateral wing (black arrow) of PSU retractor to aid for watching and cutting transverse carpal ligament (A) as compared with Senn retractor (B).

**Figure 4:**
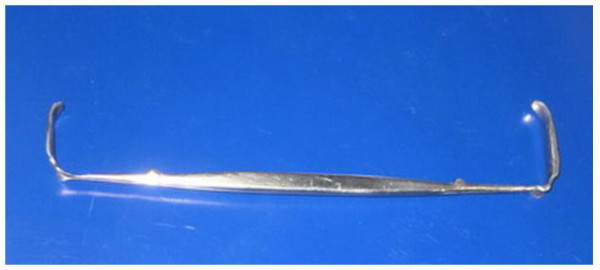
**Two sizes in blade of PSU retractor**. Demonstrated two different sizes in blade of PSU retractor.

The design concept and hand drawn illustrations were drafted and discussed with a design engineer before creating 3-dimensional model using the SolidWorks program (SolidWorks^® ^Office Premium 2007 SP3.1, SolidWorks Corporation, Concord, MA, USA). The 3-D models were popularly use in engineering-tool design because it is easily to readjust and modified. It can be connected to the rapid prototype machine for produce similar prototype. We developed an instrument we called the PSU retractor (Figure 1) from modifications to the standard Senn retractor. The main concepts were to improve visualization, protect the soft tissue around the carpal tunnel area and to allow a better passage for a cutting instrument compared with the Senn retractor. The main modified parts were the bilateral notch, a curved blade with bilateral wings, longer and tapered ends, and two different sizes of bilateral arms. The bilateral notch (Figure 2) was designed to prevent slippage and to reduce the shearing force between the blade and skin. The curved blade was designed to improve the surgeon's visual length field (Figure 3). The longer and more tapered blade compared with the Senn retractor aimed to make the retractor application easier (Figure 2). The two different sizes for the bilateral arms were designed to be more appropriate for different carpal tunnel sizes (Figure 4), rather than the 'one size fits all'. Finite element analysis was also performed to evaluate the mechanical properties of both the Senn and PSU retractor.

### Comparison of maximum visual length

**Figure 5:**
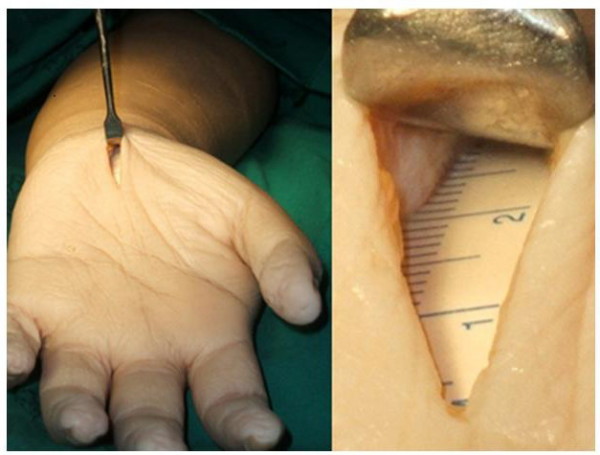
**The maximum visual length**. The maximum visual length was defined as the farest distance seen on scale of the ruler from the proximal edge of skin incision.

This study was approved from ethic committee of Faculty of Medicine, Prince of Songkla University. An amputated arm from a 30-year-old woman who had suffered synovial sarcoma at the shoulder was used to test the maximum visual length of both the Senn and PSU retractors. A mini-incision (18 mm long) was made along the longitudinal palmar crease, 3 centimetres distal to the transverse wrist crease. An incision of 10 mm was made at the palmaris longus fascia to expose the transverse carpal ligament. A flexible tiny ruler (5 mm wide and 10 cm long (Securline^® ^Surgical Skin Marker, Daigger & Company, Inc., Chicago, IL, USA)) was inserted through the carpal tunnel to measure the visual length and was fixed with nylon 3-0. We used two surgical lights (STERIS^® ^SQ 140 Surgical light, SOMA Technology, Inc. Cheshire, CT, USA) angled at 30° pointed at the wrist at a distant of 2 meters. Eight assessors (3 second-year orthopaedic residents, 4 forth-year orthopaedic residents, 1 fifth-year orthopaedic residents and 2 orthopaedic surgeons) were gathered to test the usefulness of the prototype PSU retractor. Each assessor assessed the maximal visual length of both the Senn and PSU retractors in four separate trials. The maximum visual length was defined as from the proximal edge of the skin incision to the last visible mark on the ruler (Figure 5). All assessors were also requested to comment on their level of satisfaction and comfort with the prototype using a visual analogue scale.

### Statistical analysis

All analyses were performed using SPSS software (Software Package for Social Sciences, version 13; SPSS Inc., Chicago, IL). The student t-test was used for comparison of maximal visual lengths and satisfaction scores between the Senn and PSU retractors. All continuous data was tested for normal distribution using Kolmogorov-Smirnov test.

## Results

### Instrument development

By using 3-dimensional computer design software (SolidWorks^® ^Office Premium 2007 SP3.1), a 3-dimensional computer model can be constructed (details in Figures 1A and 1B) which could show the designed object from any perspective, and rotate it, including oblique views, cross sections, perspective or isometric views. The main differences between the new PSU retractor and the Senn retractor are shown in Figures 2, 3 and 4.

### Finite element analysis

**Figure 6:**
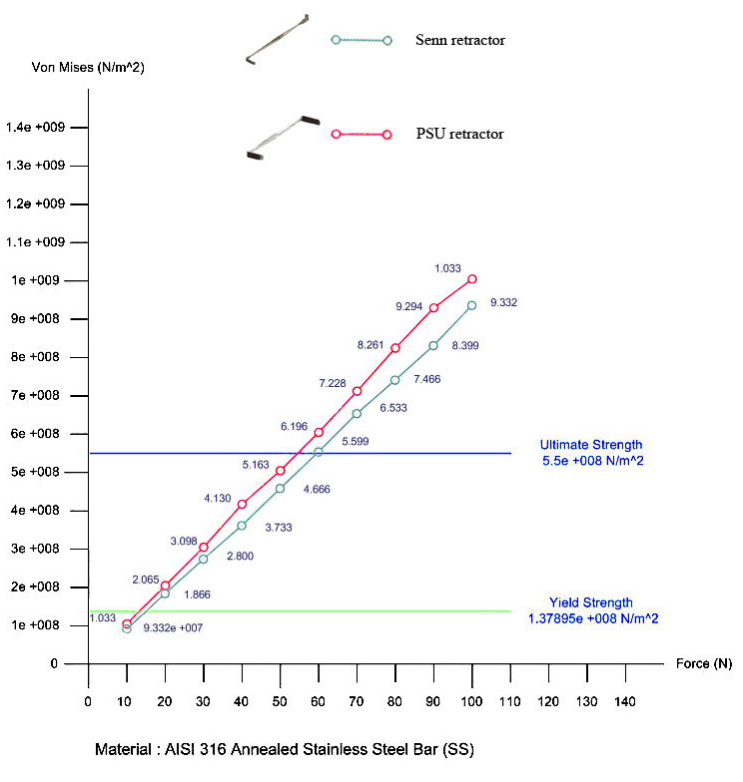
**The graph of the yield strength and the ultimate strength of both Senn and PSU retractor**. The yield strength and the ultimate strength of both Senn and PSU retractor were comparable from finite element analysis.

With 3-D computer design software (SolidWorks^® ^Office Premium 2007 SP3.1), the Finite element analysis revealed that both the PSU and Senn retractors had comparable yield strength and ultimate strength. Both retractors could tolerate force greater than 50 Newtons before mechanical failure (Figure 6).

### Visual length test

With the maximum visual length of the PSU retractor from 8 assessors, the mean maximum visual length was 47.7 (8.1) mm, which was statistically significantly better (p value less than 0.05) than the Senn retractor (39.2 (6.5) mm).

### Satisfaction of instruments

After using the instruments, all assessors weigh the level of satisfaction score by visual analogue scale. The mean level of satisfaction score of the PSU retractor was 8.5 (0.5), which was statistically significant higher (p value less than 0.05) than the satisfaction level the doctors expressed with the Senn retractor (6.4 (1.2)).

## Discussion

We have developed a new instrument called the PSU retractor to be used for mini-incision carpal tunnel release using a computer program popular with engineers. Our preliminary results showed that this new retractor significantly improved maximal visual length compared to the currently popular Senn retractor during surgical release of the deep transverse carpal ligament, and finite element analysis showed that the new retractor had comparable mechanical properties comparable to the Senn retractor. Most of the assessors who tested the instrument were satisfied with it.

Nowadays, there are a number of 3-dimentional computer-aided design programs available on the market such as SolidWorks and AutoCAD. These programs can be used to model virtually any images or designs and also have some mechanical testing applications. This is quite useful for orthopaedic surgeons, who use many sophisticated instruments in their practice but are always looking for better ones to improve their patient outcomes. In this study, we used the SolidWorks program to design a new retractor, because this is an easy program to learn and easy to adapt to different uses. Furthermore, additional options of this program include modified surfacing, finite analysis, flow analysis, mechanical motion and animation. Our study confirmed its utility in the design and modelling of our new instrument.

Mini-incision carpal tunnel operations using assisting tools such as the Indiana Tome, the KnifeLight and the "Safeguard" system have been increasingly used because they are simpler procedures with less scarring, less postoperative pain and faster recovery [9-12]. Recently, Chapman, et al.[8] reported a case of a complete median nerve transection from a carpal tunnel release using the Indiana Tome. Abouzahr, et al [7] also demonstrated 1 palmar arch injury from 28 cadavers. Our new retractor improves visual length, which should be useful in reducing or preventing potential neurovascular complications from blind mini-incision techniques.

Carpal tunnel syndrome is the most common disease in orthopaedic practice. More than 200,000 operations of carpal tunnel surgery are reported each year in the US, at a cost per procedure of between 5,500 and 11,900 US dollars [13] with a total of more than 1 billion US dollars [14-16]. The new PSU instrument could have a significant financial benefit as it reduces the need for the current costly special cutting tools or sophisticated endoscopic procedures [17-19]. The carpal tunnel release mini-surgery can also be performed under local anesthesia, which will again reduce both the cost and risk of anesthesia.

However, our study is only a preliminary report using one carpal tunnel with a group of assessors, so further studies need to be done in actual clinical settings to compare the new instrument with other instruments and in different settings for both efficacy and safety.

## Conclusion

The PSU retractor was developed using a CAD program (SolidWorks) and demonstrates promising properties which should be useful in mini-incision carpal tunnel release procedures. However further clinical studies are required to confirm our results.

## Competing interests

The authors declare that they do not have a conflict of interest. The design presented in the manuscript is patented. However, the authors have no interest, reimbursement, arrangement or affiliation that could be interpreted as a conflict of interest pertaining to this manuscript.

## Authors' contributions

SW contributed to the original idea, concept, modelling design, and drafting of the manuscript. PS and VY contributed to data extraction, interpretation of the maximum visual length and satisfaction scores. BT contributed to the concept, data extraction, statistical analysis and critically revised the manuscript. EW contributed to creating the idea and modelling design. All authors read and approved the final manuscript.

## Pre-publication history

The pre-publication history for this paper can be accessed here:


